# Supplementary material for: The interactome of intact mitochondria by cross-linking mass spectrometry provides evidence for coexisting respiratory supercomplexes
Source: Mol Cell Proteomics. 2017 Dec 8;17(2):216–32. doi: 10.1074/mcp.RA117.000470 (PMC5795388; doi:10.1074/mcp.RA117.000470)
Supplement: Supplemental Data [file supp_RA117.000470_133922_0_supp_23978_fzffwf.zip › spectra_annotation/mito_DR_spectra_annotation/436-1-11-1-16-1.pdf]

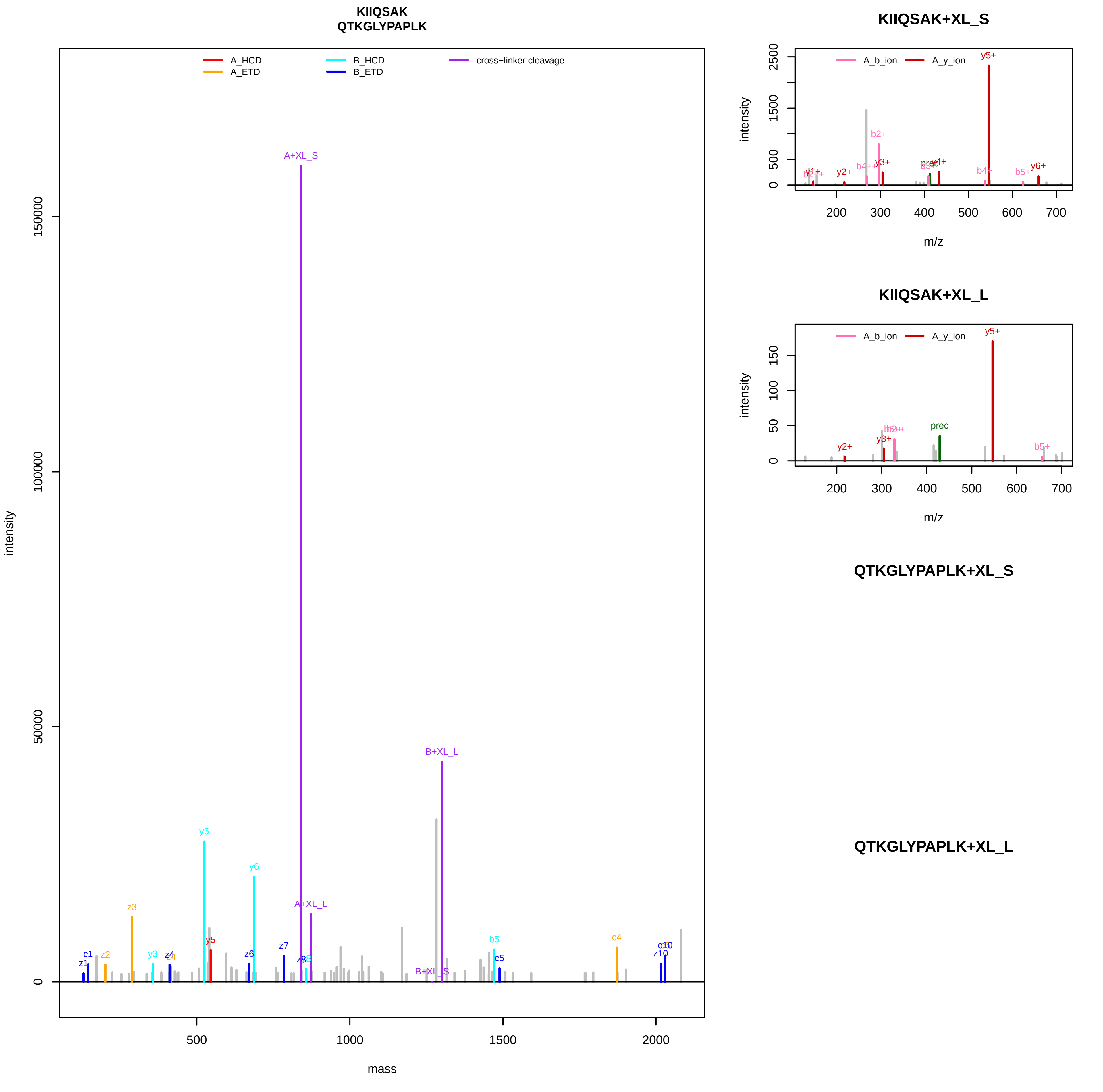
KIIQSAK
QTKGLYPAPLK
A_HCD
A_ETD
B_HCD
B_ETD
cross−linker cleavage
A+XL_S
B+XL_L
A+XL_L
B+XL_S
y5
y6
z3
z2
c1
z1
y3
z4
z6
z7
z8
b5
c5
c4
c10
z10
intensity
mass
0
50000
100000
150000
500
1000
1500
2000
KIIQSAK+XL_S
A_b_ion
A_y_ion
y5+
b2+
y3+
b4++
prec
y4+
y1+
y2+
b4+
b5+
y6+
m/z
KIIQSAK+XL_L
QTKGLYPAPLK+XL_S
QTKGLYPAPLK+XL_L
